# P5L mutation in Ank results in an increase in extracellular inorganic pyrophosphate during proliferation and nonmineralizing hypertrophy in stably transduced ATDC5 cells

**DOI:** 10.1186/ar2072

**Published:** 2006-10-26

**Authors:** Raihana Zaka, David Stokes, Arnold S Dion, Anna Kusnierz, Fei Han, Charlene J Williams

**Affiliations:** 1 Division of Rheumatology, Department of Medicine, Thomas Jefferson University, Philadelphia, PA 19107, USA; 2 College of Graduate Studies, Thomas Jefferson University, Philadelphia, PA 19107, USA

## Abstract

Ank is a multipass transmembrane protein that regulates the cellular transport of inorganic pyrophosphate. In the progressive ankylosis (*ank*) mouse, a premature termination mutation at glutamic acid 440 results in a phenotype characterized by inappropriate deposition of basic calcium phosphate crystals in skeletal tissues. Mutations in the amino terminus of ANKH, the human homolog of Ank, result in familial calcium pyrophosphate dihydrate deposition disease. It has been hypothesized that these mutations result in a gain-of-function with respect to the elaboration of extracellular inorganic pyrophosphate. To explore this issue in a mineralization-competent system, we stably transduced ATDC5 cells with wild-type Ank as well as with familial chondrocalcinosis-causing Ank mutations. We evaluated the elaboration of inorganic pyrophosphate, the activity of pyrophosphate-modulating enzymes, and the mineralization in the transduced cells. Expression of transduced protein was confirmed by quantitative real-time PCR and by ELISA. Levels of inorganic pyrophosphate were measured, as were the activities of nucleotide pyrophosphatase phosphodiesterase and alkaline phosphatase. We also evaluated the expression of markers of chondrocyte maturation and the nature of the mineralization phase elaborated by transduced cells. The cell line expressing the proline to leucine mutation at position 5 (P5L) consistently displayed higher levels of extracellular inorganic pyrophosphate and higher phosphodiesterase activity than the other transduced lines. During hypertrophy, however, extracellular inorganic pyrophosphate levels were modulated by alkaline phosphatase activity in this cell system, resulting in the deposition of basic calcium phosphate crystals only in all transduced cell lines. Cells overexpressing wild-type Ank displayed a higher level of expression of type X collagen than cells transduced with mutant Ank. Other markers of hypertrophy and terminal differentiation, such as alkaline phosphatase, osteopontin, and *runx2*, were not significantly different in cells expressing wild-type or mutant Ank in comparison with cells transduced with an empty vector or with untransduced cells. These results suggest that the P5L Ank mutant is capable of demonstrating a gain-of-function with respect to extracellular inorganic pyrophosphate elaboration, but this effect is modified by high levels of expression of alkaline phosphatase in ATDC5 cells during hypertrophy and terminal differentiation, resulting in the deposition of basic calcium phosphate crystals.

## Introduction

The pathologic deposition of calcium pyrophosphate dihydrate crystals in the joints of patients with familial chondrocalcinosis is associated with mutations in ANKH (for a review, see [1]). The ANKH gene is the human homologue of the gene responsible for progressive ankylosis in a naturally occurring mutant mouse [2]. The product of the *ank*/ANKH gene appears to regulate the transport of inorganic pyrophosphate (PPi) through the cell membrane. Sohn and colleagues originally observed high expression of Ank in the hypertrophic growth plate [3]. Interestingly, high levels of ANKH expression have also been found in osteoarthritic cartilage and in cartilage from patients with calcium pyrophosphate dihydrate deposition (CPPD), particularly in areas of cartilage tissue that are populated with hypertrophic-like chondrocytes [4-6]. While these observations suggest that Ank plays a role in the pathological mineralization of cartilage, Wang and colleagues [7] have shown that the protein also has an important role in the physiological mineralization of the chick tibial growth plate by demonstrating that increased Ank activity led to decreased levels of extracellular PPi resulting from the concomitant upregulation of alkaline phosphatase (AP) expression.

To characterize the role of wild-type Ank and its mutants in the regulation of PPi transport *in vitro*, we stably transduced ATDC5 cells with wild-type Ank and three missense mutations we have reported in families with familial chondrocalcinosis. We chose to only modestly overexpress wild-type and mutant Ank in order to create a stable dominant-negative environment in which to evaluate PPi elaboration, as well as the activity of two enzymes that are critical to the fate of PPi generation: nucleotide pyrophosphohydrolase phosphodiesterase (NPP) and AP. These studies were performed in ATDC5 cells to take advantage of the fact that these well-characterized cells are fully mineralization competent [8] and are amenable to stable transfection. Furthermore, the use ofATDC5 cells permitted us to address some critical issues concerning the biochemical and physiological impact of overexpression of wild-type Ank, and the expression of mutant forms of Ank, on the course of chondrogenesis.

## Materials and methods

### Cell culture, proliferation assays, and gene expression studies

ATDC5 cells [9] (3 × 10^4 ^cells/35 mm dish) were maintained in DMEM/Ham's F-12 (1:1) containing 5% fetal bovine serum, 2 mM L-glutamine, 10 μg/ml human transferrin, and 3 × 10^-8 ^M sodium selenite (maintenance medium), or the cells were differentiated, without passage, in the same medium supplemented with 10 μg/ml insulin (chondrogenic medium). The media were changed every other day. Proliferation of untransduced cells or transduced cells was monitored using the cell proliferation agent WST-1 (Roche, Indianapolis, IN, USA). Cells were propagated in 96-well plates at the same cell density as described above. Following addition of WST-1 reagent, the optical density was read at 450 nm. The background was determined by assay of clean media collected at equivalent time points.

For studies of mineralization in ATDC5 cells, at day 21 of culture, α-MEM medium containing 5% fetal bovine serum 2 mM glutamine, 10 μg/ml human transferrin, 3 × 10^-8 ^M sodium selenite, and 10 μg/ml insulin was added to the cell cultures without passage of cells. The concentration of CO_2 _was also switched to 3%, as previously described [8]. The medium was replaced every other day. For measurements of mineral content by Fourier transform IR analyses, cell layers were washed with phosphate-buffered saline, scraped into 0.1 M ammonium bicarbonate solution (pH 8.5), pelleted, and lyophilized.

For experiments in which the constitutive expression of *ank *was assessed, cells were incubated in parallel cultures containing maintenance medium and chondrogenic medium for a period of 21 days. Cells were harvested at the times indicated above for poly A+ RNA isolation using the Micro-FastTrack 2.0 kit according to manufacturer's specifications (Invitrogen, Carlsbad, CA, USA). For cDNA synthesis, 150 ng mRNA was reverse transcribed using the ThermoScript RT-PCR system (Invitrogen). The resultant cDNA was utilized for quantitative RT-PCR, using β-actin as standard. The primers used to amplify *ank *were sense primer 5' -cttctagcagggtttgtggg-3' (in exon 11 of the transcript) and antisense primer 5' -tcgtctctttcctcctcctc-3' (in the 3' -untranslated region; product = 166 bp). Thermocycling was performed in a MyIQ thermocycler (Biorad, Hercules, CA, USA) using a reaction mix containing syber green. A melting curve was performed for each PCR cycling reaction to ensure recovery of a single syber green fluorescing species in the reaction product. The fold changes of steady-state RNA levels were determined by the formula 2^-ddCt^, where ddCt = dE – dC (dE = Ct_exp _– Ct_actin _and dC = Ct_contl _– Ct_actin; _dE = delta experimental, dC = delta control, Ct = cycling threshold).

### Preparation of FLAG-tagged Ank constructs and transient transfection with FLAG-tagged constructs

The wild-type sequence of murine *ank *was used for both transient and stable cDNA constructs of *ank*, and all mutations in *ank *were prepared in the context of the mouse cDNA sequence. *Ank *cDNA was subcloned into a pcDNA I vector (Invitrogen) containing a FLAG sequence at the amino terminus of the multiple cloning site. To generate an inframe FLAG tag, the stop codon of each *Ank *cDNA – wild type and the proline position 5 to leucine (P5L), proline position 5 to threonine (P5T), and methionine position 48 to threonine (M48T) mutants – was ablated by site-directed mutagenesis and the FLAG tag was added to the 3' end of each cDNA by the PCR. The integrity of each construct was confirmed by direct sequence analysis of the entire cDNA-FLAG insert.

For transfection with pcDNA I/*ank*-FLAG cDNA constructs, ATDC5 cells were transfected with wild-type and mutant constructs (1 μg plasmid DNA/ml medium) in the presence of FuGene 6 reagent (Roche) at a ratio of 1 μg plasmid DNA:2.5 μl FuGene 6 reagent. A construct containing the *lacZ *gene was prepared as a control for transfection efficiency using the same *lacZ*:FuGene6 ratio. After 48 hours of culture in maintenance medium, cells were fixed with 4% *p*-formaldehyde and were immediately processed for immunohistochemistry using a mouse polyclonal anti-FLAG M2 antibody (Stratagene, La Jolla, CA, USA). and goat anti-mouse secondary IgG antibody conjugated to Alexa fluor 488 (Molecular Probes, Eugene, OR, USA). Parallel chamber slides were incubated in secondary antibody only. Cells were mounted with VectaShield (Vector Laboratories, Inc., Burlingame, CA, USA) mounting medium with propidium iodide for fluorescent detection of double-stranded DNA, and the cells were then visualized on a Zeiss LSM510 Mera confocal microscope (Carl Zeiss MicroImaging, Thornwood, NY, USA) using a 63x lens.

### Preparation of retroviral *Ank *constructs and stable transduction of ATDC5 cells

For the preparation of stable transfections via retroviral transduction, *ank *cDNA wild-type and mutant constructs, prepared in the context of the mouse cDNA sequence, were subcloned into the pLNCX expression vector (Clontech, Palo Alto, CA, USA) and were packaged as directed by the manufacturer. Virus-containing medium was directly added to ATDC5 cells that had been plated to 80% confluence in maintenance medium 24 hours prior to infection. After 24 hours cells were subjected to selection with the neomycin resistance reagent G418 (350 μg G418/ml media) for 2 weeks (media were changed every 4 days). Approximately 10% of cells survived selection and were expanded at low density in 100 μg G418/ml media and were further subjected to clonal selection. Cells were expanded in the presence of G418 to ensure retention of the transduced cDNAs, and were eventually harvested for mRNA isolation to evaluate relative expression of endogenous and transduced cDNAs.

DNA was also isolated and used in Southern blot analyses to confirm the clonality of the cell lines. Genomic DNA was cleaved with *Xba*I (an enzyme that cleaves only once in the pLNCX vector in the 3' long terminal repeat region), blotted, and probed with a PCR product derived from the cytomegalovirus promoter region of the pLNCX vector to exclude detection of endogenous *ank *sequences.

### Detection of expression of transduced Ank in ATDC5 cells by real-time PCR

Real-time PCR was used to measure levels of both endogenous and transduced *ank *transcripts in clonally selected populations. For detection of transduced wild-type or mutant *ank *transcripts, PCR primers were derived from sequences between exons 11 and 12 of the *ank *cDNA (sense primer, 5' -ggtttgtgggagaatctacc-3'); the antisense primer was derived from the pLNCX vector (5' -ccccctttttctggagacta-3' ; product size = 265 bp). For detection of endogenous *ank *transcript, the primers described earlier in Materials and methods were used. The ratio of PCR products was determined by comparison of the ddCt values for the endogenous transcript divided by the ddCt for the transduced transcript, as previously described. For each cloned transductant, four separate clones expressing a 1:1 ratio of endogenous to transduced transcript were independently evaluated for Ank protein expression.

### ELISA determination of Ank protein expression in stably transduced cells

Cells were harvested, in the presence of protease inhibitor, from confluent cultures of transduced cells and were disrupted by rapid freeze/thaw with final dispersion through an 18-gauge needle. Protein was quantitated by the Bradford Coomassie assay (Pierce, Rockford, IL, USA), using bovine serum albumin as the standard.

Polyclonal anti-Ank antisera were generated (Cocalico, Biologicals Inc., Westville, PA, USA) in Leghorn chickens against a synthetic peptide immunogen derived from the Ank carboxy terminus conjugated to keyhole limpet hemocyanin, as previously described [2] for the preparation of an Ank-specific antiserum. Ammonium sulfate-precipitated chicken antibody derived from blood sera was delipidated with *n*-butanol/diisopropyl ether in a 40:60 (vol/vol) ratio [10].

ELISA procedures relevant to the determination of antibody or antigen titers using a twofold dilution series have been described [11]. Primary antibody binding to antigens was detected with an affinity-purified, peroxidase-conjugated donkey anti-chicken IgY (Jackson Immunoresearch Laboratories, Inc., West Grove, PA, USA) at a dilution of 1:5,000. The secondary antibody was then quantitated with a chromogenic substrate, *o*-phenylenediamine, and the optical densities at 490 nm were recorded with a microplate reader (Opsis MR Microplate Reader; Thermo/Labsystems, Waltham. MA, USA) using Revelation Quicklink software (Dynex Technologies, Chantilly, VA, USA).

### Intracellular and extracellular inorganic pyrophosphate assays

For studies of extracellular inorganic pyrophosphate (ePPi) and intracellular inorganic pyrophosphate (iPPi) elaboration in cells undergoing differentiation, as well as for measurements of AP and NPP activities, and measurement of expression of markers of hypertrophy (see below), cells were cultured in differentiation medium until 24 hours prior to assay. At this time, media for cells to be assayed were refreshed with maintenance medium (which does not contain insulin).

For assay of iPPi, cells were harvested heated at 65°C for 1 hour and were lysed in lysis buffer containing 1% Triton X-100, 1.6 mM MgCl_2_, and 0.2 M Tris, pH 8.0. For assay of ePPi, media were cleared of cellular debris and were diluted 1:2 in lysis buffer. PPi levels were evaluated by the enzymatic procedure of Lust and Seegmiller [12], as modified by Johnson and colleagues [13], where PPi is determined by differential absorption on activated charcoal of UDP-D- [6-^3^H]-glucose from the reaction product 6-phospho- [6-^3^H]-gluconate. All assay results were normalized versus DNA concentration using a Pico Green assay of double-stranded DNA (Molecular Probes).

### Assays of alkaline phosphatase and nucleotide pyrophosphohydrolase activity

The diethanolamine enzymatic assay (Sigma, St Louis, MO, USA) was used to measure the AP (EC 3.1.3.1) activity [14]. Cells were disrupted, were reacted with substrate solution containing a final concentration of 15 mM *p*-nitrophenyl phosphate in the presence of 1 M diethanolamine and 0.5 mM MgCl_2_, pH 9.8, and the optical densities of the reaction product (*p*-nitrophenol) were determined at 405 nm at time 0 and at 1 and 2 minutes after the start of the reaction. The AP activity was normalized to the total protein concentration of diluted cell lysates as determined by use of the BCA Protein assay (Pierce). Inhibition of AP activity was performed with the addition of 3 μM levamisole, which was added to the cell culture medium for 72 hours prior to harvesting for AP activity measurements.

For the assay of NPP (EC 3.6.1.8, EC 3.1.4.1) activity, 1 mM *p*-nitrophenyl-thymidine 5' -monophosphate (Sigma) was used as substrate in a reaction to which 5 μl cell lysate was added [13]. A standard curve consisting of *p*-nitrophenol in 50 mM Hepes-buffered DMEM and 1.6 mM MgCl_2 _was also prepared. Reactions were terminated by the addition of 55 μl of 0.1 M NaOH, and optical densities were determined at 405 nm. Final sample comparisons were expressed as units per milligram of total protein.

### Expression of markers of chondrocyte maturation and terminal differentiation

To monitor the differentiation of transduced cells, poly A+ RNA was used in real-time RT-PCR to detect the expression of type II collagen (*col2a1*), of *sox9*, and of type X collagen (*col10a1*). Primers for these transcripts were as follows: *col2a1*, sense primer 5' -gagggccaggaggtcctctgg-3' and antisense primer 5' -tcgcggtgagccatgatccgc-3' (product size = 177 bp); *col10a1*, sense primer 5' -taccacgtgcatgtgaaagg-3' and antisense primer 5' -ggagccactaggaatcctga-3' (product size = 236 bp); and *sox9*, sense primer 5' -agt tga tct gaa gcg aga ggg-3' and antisense primer 5' -cct ggg tgg ccg ttg ggt ggc-3' (product size = 169 bp).

Expression levels of additional markers of the hypertrophic phenotype were measured to evaluate the impact of *ank *mutants on hypertrophy in transduced ATDC5 cells. These additional markers included osteopontin (sense primer 5' -cac atg aag agc ggt gag tct-3', antisense primer 5' -atc gat cac atc cga ctg atc-3' ; product size = 198 bp) and *runx2 *(*cbfa1*; sense primer 5' -atggcactctggtcaccgtc-3', antisense primer 5' -cctgaggtcgttgaatctcg-3' ; product size = 110 bp).

The fold changes of steady-state RNA levels in *ank*-transduced cells compared with cells transduced with empty vector only were determined as previously described. Reactions were performed in triplicate and were repeated twice.

### Statistical methods

Data are presented as the mean ± standard deviation. The statistical significance was identified using the unpaired, two-tailed Student *t *test, unless otherwise indicated in the figure legend (*P *values reported in the figure legends). All assays were performed at least in triplicate; see figure legends for the exact number of replicates performed.

## Results

### Expression of endogenous *ank *in ATDC5 cells

Before transduction studies were performed, we determined the endogenous expression of *ank *in the cell line during a 21-day course of chondrogenesis, prior to the entry of cells into hypertrophy. Cells were plated in the presence or absence of the chondrogenic promoter insulin [9] and mRNA was isolated for determination of *ank *expression., The expression of *ank *after day 3 of culture was consistent throughout the proliferation stage in the untransduced cells regardless of the insulin treatment regimen (data not shown).

### Localization of mutant Ank molecules to the cell membrane

**Figure 1:**
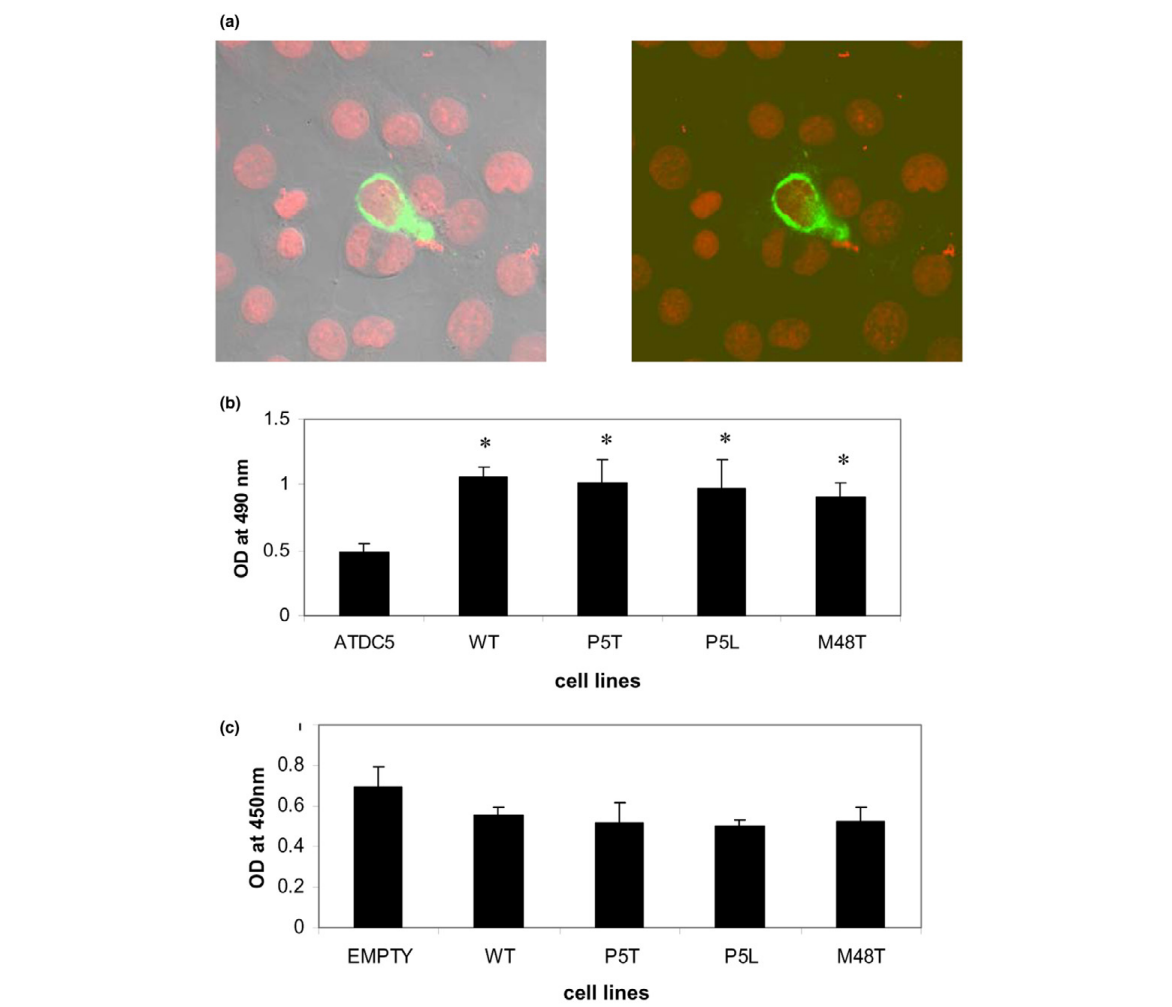
Transfection of ATDC5 cells with wild type and mutant Ank. **(a) **Confocal microscopy of ATDC5 cells transiently transfected with M48T mutant cDNA. Left panel is phase-contrast image of right panel. All transfected cells showed a similar pattern of plasma cell membrane staining, indicating that even mutant Ank molecules were able to translocate to the plasma cell membrane. **(b) **ELISA assay results showing comparative levels of Ank protein expression in ATDC5 cell lysates versus lysates of independent clones of ATDC5 cells transduced with various *ank *constructs. All cells express endogenous Ank protein, but transduced cells also express protein derived from expression of transduced constructs. Data represent quadruplicate assays and are representative of results obtained from other independent clones for each transductant. *P ≤ 0.05. **(c) **WST-1 proliferation assay at day 7 of culture in ATDC5 cells transduced with empty vector and with various wild-type (WT) or mutant *ank *constructs. At days 7, 14, and 21, 7.5 μl WST-1 reagent was added directly to 150 μl cell medium and incubated for 1.5 hours at 37°C in 5% CO_2_. Results at all time points consistently exhibited no significant differences in the proliferation of mutant-transduced cells compared with untransduced cells or cells transduced with empty vector. *n *= 3.

To confirm that the mutant Ank gene products could appropriately translocate to the cell membrane as has been observed for wild-type Ank [2], we transiently transfected FLAG-tagged mutant and wild-type *ank *constructs into ATDC5 cells. The mutant *ank *constructs were three missense mutations of ANKH that occurred in four unrelated CPPD disease families, as we previously described [15-17], and whose sequence and sequence contexts were fully conserved in the murine sequence. The missense mutations included the P5T and P5L substitutions, occurring at positions +13 bp and +14 bp of the Ank cDNA, respectively, and the M48T substitution, occurring at position +143 of the *ank *cDNA. Cells expressing mutant Ank molecules exhibited localization identical to that seen for wild-type Ank. Figure 1a demonstrates the localization of one mutant Ank molecule: the M48T mutant. In all cases, expressed Ank molecules could be visualized at the cell surface by confocal microscopy.

### Selection and characterization of clonal populations of ATDC5 cells expressing wild-type and mutant Ank

To achieve moderate levels of mutant *ank *expression in ATDC5 cells that were comparable with expression of the endogenous transcript, we chose to subject our transduced cells to a further round of selection using limiting dilution. Clonality was confirmed by Southern blot analysis (data not shown), and mRNA was isolated and subjected to real-time RT-PCR with primers specific for either the transduced *ank *transcripts or the endogenous *ank *transcripts. The clones were then analyzed for both endogenous and exogenous *ank *transcript levels, and clones that exhibited a 1:1 ratio of endogenous transcript to transduced transcript were selected for further analysis. For each wild-type or mutant construct, four independent clones exhibiting a 1:1 ratio of transduced to endogenous transcript were assayed for protein expression.

To ensure that transduced cells were expressing gene product derived from the transduced cDNAs, we first considered the possibility of adding a tag to the transduced cDNAs to follow their expression and translation into protein. We ultimately wished to use the transduced cell lines for determination of PPi levels, however, and we were acutely aware of the fact that minor perturbations in the structure of the Ank protein can have a major impact on Ank function [2,15,16,18,19]. We therefore chose to monitor the production of total Ank protein by a quantitative ELISA assay using a peptide-directed polyclonal antibody that was capable of reacting to exposed (that is, nontransmembrane) epitopes following cell disruption. We expected clones expressing a 1:1 ratio of endogenous *ank *transcript to transduced *ank *transcript to produce approximately twice the amount of Ank protein as cells that expressed endogenous *ank *transcript only. ELISA assays were performed on the cell lines, and the results indicated that clones transduced with wild-type or mutant *ank *expressed approximately twice the level of Ank protein as observed in the original, untransduced cells (Figure 1b).

To determine whether transduction of wild-type and mutant *ank *constructs affected proliferation of transduced cells, we determined the proliferation and cell viability based upon the cleavage of the tetrazolium salt, WST-1, by mitochondrial dehydrogenases in viable cells. At days 7, 14, and 21 of assay there were no significant statistical differences in proliferation and viability in cells transduced with wild-type *ank *or mutant *ank *compared with cells transduced with empty vector (Figure 1c). These results demonstrated that the transduction of *ank *did not affect the ability of cells to proliferate normally.

### Levels of intracellular and extracellular inorganic pyrophosphates in transduced ATDC5 cells

**Figure 2:**
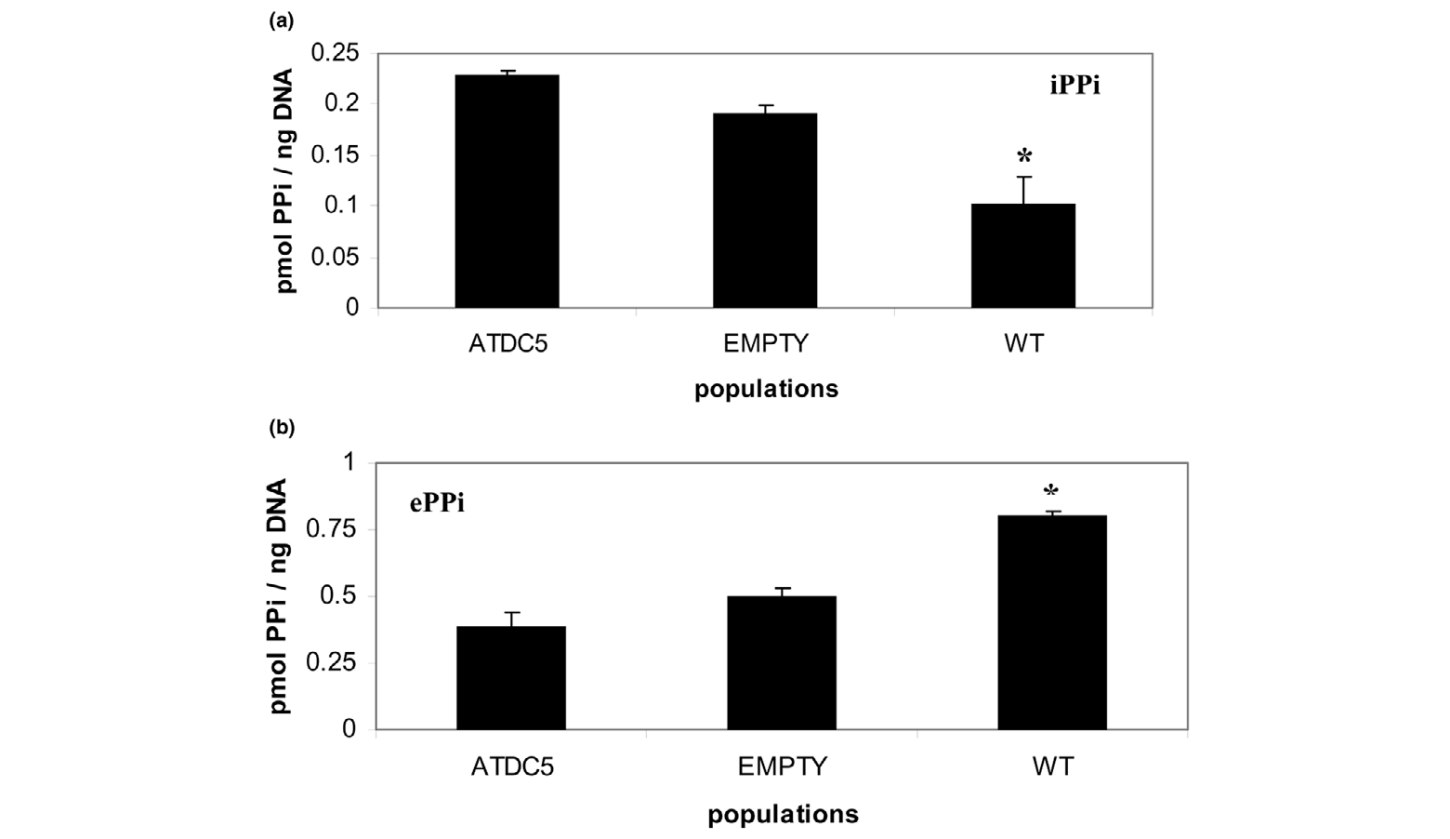
Extracellular and intracellular inorganic pyrophosphates in ATDC5 cells transduced with wild-type vector before clonal selection. Cells were assayed at day 14 of chondrogenesis. **(a) **Consistent with previous reports of the impact of overexpression of *ank *on levels of intracellular inorganic pyrophosphate (iPPi) and extracellular inorganic pyrophosphate (ePPi), cells transduced with wild-type (WT) *ank *demonstrate a decrease in levels of iPPi when compared with cells transduced with pLNCX empty vector or with untransduced ATDC5 cells. **(b) **Concomitantly, cells transduced with WT *ank *exhibit an increase in ePPi levels when compared with cells transduced with pLNCX vector only (empty) or with untransduced ATDC5 cells. *n *= 6; *P ≤ 0.05. PPi, inorganic pyrophosphate.

The levels of PPi are variably affected by many growth factors and cytokines (for a review, see [20]). Because of the reported effects of insulin-like growth factor 1 on the elaboration of PPi in chondrocytes [21,22], we chose to eliminate exogenous insulin from the medium used for the studies of PPi levels in transduced ATDC5 cells for 24 hours prior to assay. We first evaluated the impact of overexpression of *ank *in day 14 (proliferation phase) cells transduced with wild-type *ank*, but prior to clonal selection. Overexpression of *ank *resulted in a statistically significant decrease in iPPi (Figure 2a) and a concomitant increase in ePPi (Figure 2b), as has been previously reported in COS cells and in bovine chondrocytes [2,6].

**Figure 3:**
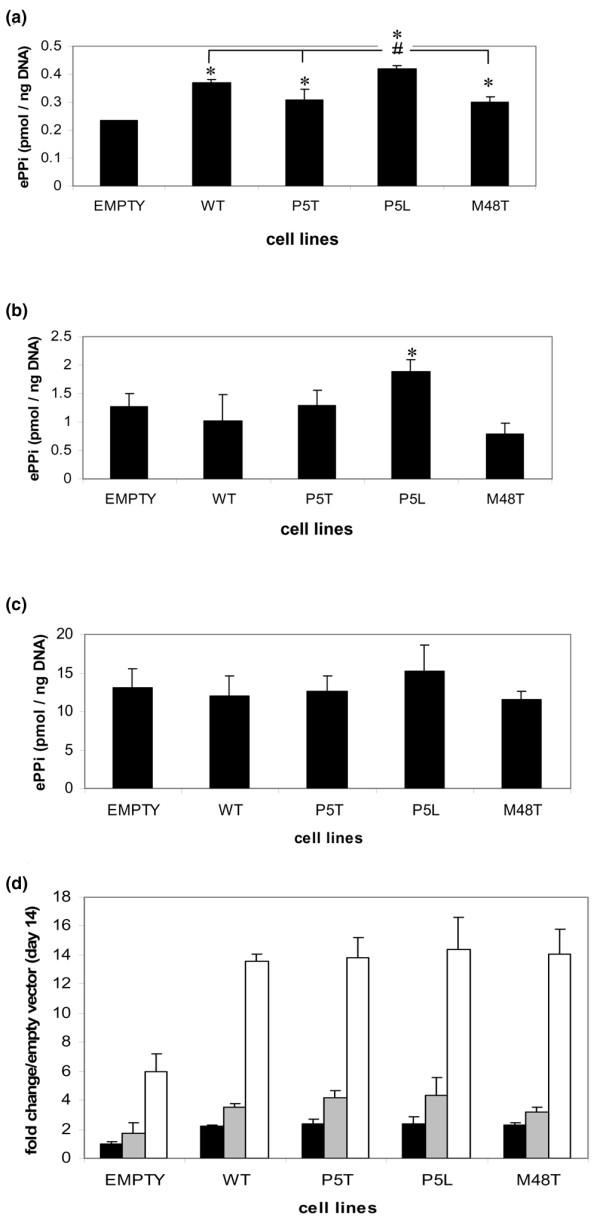
Expression of Ank and generation of extracellular PPi in transduced ATDC5 cells. **(a) **Extracellular inorganic pyrophosphate (ePPi) levels in various ATDC5 clonal cell lines at day 14 of chondrogenesis. Empty, uncloned ATDC5 cells transduced with pLNCX vector only; WT, wild-type *ank*. *Significance of ePPi levels of WT and mutant Ank-transduced cell lines versus cells transduced with empty vector only. #Significance of P5L ePPi levels versus cells transduced with WT Ank or mutant ank constructs, as indicated. **(b) **ePPi levels in various ATDC5 clonal cell lines at day 28 (nonmineralizing hypertrophy) of chondrogenic differentiation. **(c) **ePPi levels in various ATDC5 clonal cell lines at day 35 (mineralization) of chondrogenic differentiation. WT, P5T, P5L, and M48T are independent clonal cell lines of stably transduced ATDC5 cells exhibiting a 1:1 transcript level ratio of endogenous *ank *to transduced *ank *and twice as much Ank protein as untransduced cells. At least three independent clones for each cell line were evaluated; results presented are from single clones and are representative of other clones for each cell line. Inorganic pyrophosphate levels for untransduced ATDC5 cells and empty vector were comparable. *n *= 9; *P ≤ 0.05. **(d) **The fold change in the expression of *ank *mRNA as determined by real-time RT-PCR at various times of chondrogenesis. Black bars, day 14 (proliferation); grey bars, day 28 (nonmineralizing hypertrophy); white bars, day 35 (mineralizing hypertrophy). Note increase of expression in *ank *under mineralizing conditions, which is consistent with the dramatic increase in ePPi in transduced cells at day 35 of culture.

We next examined the impact of mutations in Ank on the elaboration of ePPi in proliferating ATDC5 clonal cell lines, in nonmineralizing hypertrophic ATDC5 clonal cell lines, and in mineralizing ATDC5 clonal cell lines. The results demonstrate that, during their proliferating phase, all cells stably transduced with *ank *exhibit higher levels of extracellular PPi than cells transduced with empty vector only; however, only cells stably transduced with the P5L mutant exhibited levels of ePPi significantly greater than cells transduced with the other mutants or cells transduced with wild-type Ank (Figure 3a). These same results were observed for the P5L cell line when ePPi levels were measured at hypertrophy under nonmineralizing conditions. Also, at hypertrophy there is no statistically significant difference in ePPi elaboration among cells that were transduced with wild-type Ank and the P5T and M48T mutants in comparison with cells transduced with empty vector (Figure 3b). Finally, ePPi levels were evaluated from cells that were mineralized, and we observed a significant increase in ePPi elaboration in all transduced cells (Figure 3c).

In light of reports suggesting that Ank expression is increased in regions of cartilage undergoing terminal differentiation and mineralization, or in response to agents that induce mineralization [3,6,7], we explored the possibility that the high levels of ePPi in mineralized cells might be a function of increased Ank expression. Figure 3d illustrates that *ank *expression is roughly equivalent among the cells lines transduced with wild-type or mutant *ank *at all three stages of chondrogenesis; however, the expression of *ank *in the transductants increased as the cells progressed toward mineralized hypertrophy. Notably, there was an approximately fourfold increase in *ank *expression in the transduced cells at mineralized hypertrophy compared with that in nonmineralized hypertrophic cells. As illustrated in Figure 3c, in mineralizing conditions we did not observe a statistically significant increase in ePPi in any of the mutant cell lines, including the P5L cell line, when compared with cells transduced with wild-type *ank*. Additionally, there was no statistically significant difference in the elaboration of ePPi in cells that overexpressed wild-type Ank compared with cells transduced with empty vector only.

Our observations were consistent with the previous observations of Wang and colleagues [7], although not as dramatic as those they observed. More specifically, Wang and colleagues observed that retroviral-driven overexpression of Ank in hypertrophic chondrocytes actually decreased the elaboration of ePPi, apparently due to increased activity of AP resulting from the overexpression of Ank (see below), while, as stated above, we observed that modest overexpression of Ank did not result in an increase in the elaboration of ePPi compared with cells transduced with empty vector only. Since ATDC5 cells have been shown to elaborate significant amounts of AP during their mineralization phase [8], we hypothesized that the lack of ePPi excess in cells overexpressing Ank compared with that in cells transduced with empty vector, or in the P5L mutant cell line, may be due to hydrolysis of PPi by AP. We therefore next evaluated the nature of PPi elaboration at hypertrophy and at mineralization as a function of AP activity, as well as of NPP activity.

### Alkaline phosphatase and nucleotide pyrophosphohydrolase activity activities in ATDC5 cells transduced with wild-type and mutant Ank

In the complex regulation of cellular PPi elaboration, two major enzyme systems play an important role in the generation of extracellular PPi: NPP, an ecto-enzyme that can hydrolyze nucleoside triphosphates into their monophosphate esters and PPi; and AP, an enzyme with pyrophosphatase activity [23]. We evaluated the levels of both of these enzymes in transduced cells during the proliferative phase of differentiation (day 14), the nonmineralizing hypertrophic phase of differentiation (day 28), and the mineralization phase of differentiation (day 35).

**Figure 4:**
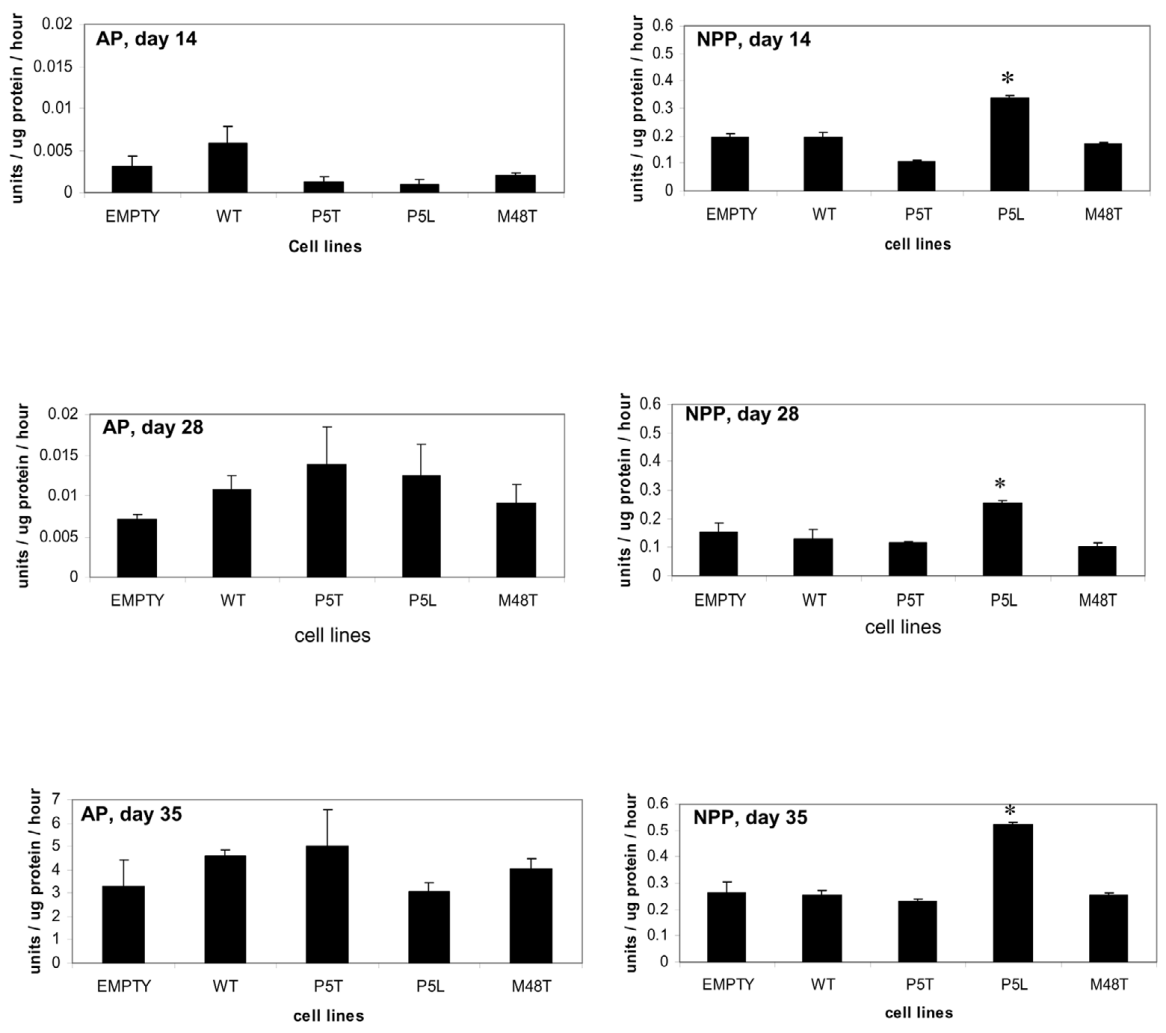
Alkaline phosphatase and nucleotide pyrophosphatase phosphodiesterase activity in transduced clones. Enzyme activities were measured in transduced clones at day 14 (proliferation), at day 28 (nonmineralizing hypertrophy), and at day 35 (mineralization). Empty, uncloned cells transduced with pLNCX vector; WT, wild-type *ank*. For alkaline phosphatase (AP) measurements, the units of enzyme were determined by first subtracting the optical density reading of a blank from diluted samples at 2 minutes. The result of this calculation was then subtracted from a similar calculation performed on samples determined at time point 0. Levels of AP are negligible at day 14 of culture and increase at day 28. Consistent with previous studies [8], AP activity is much higher in cells at day 35 of culture during mineralization. Although AP activity is higher for cells overexpressing WT *ank *compared with cells transduced with empty vector at days 28 and 35 of culture, cells expressing mutant *ank *constructs did not demonstrate AP activity that was significantly different from cells transduced with WT *ank*. Nucleotide pyrophosphatase phosphodiesterase (NPP) activity for each sample were determined by comparison with the standard curve of *p*-nitrophenol and expressed as units, where one unit is equivalent to 1 μmol substrate hydrolyzed per hour. With the exception of the cell line transduced with the P5L mutant, all transduced lines exhibited NPP activity that was comparable with cells transduced with empty vector only. AP and NPP activities for untransduced cells and empty vector were comparable. *n *= 9; *P ≤ 0.05. At least three independent clones for each cell line were evaluated; results presented are from single clones and are representative of other clones for each cell line.

AP activity was very low during the proliferative stage of ATDC5 cell chondrogenesis in all of the cell lines tested (Figure 4); however, its activity increased during both the nonmineralizing hypertrophic phase of differentiation and in mineralizing cells. Consistent with previous reports of ATDC5 cellular hypertrophy and mineralization [8], the levels of AP activity increased significantly between day 28 at nonmineralizing hypertrophy and day 35 when the cells were mineralized. Although AP levels were somewhat higher in cells overexpressing wild-type *ank *versus cells transduced with empty vector only, neither nonmineralizing hypertrophic cells nor mineralized cells that had been transduced with mutant *ank *constructs demonstrated significantly different levels of AP activity to cells overexpressing wild-type *ank *(Figure 4). The increase in AP activity in response to the modest overexpression of wild-type *ank *was not as great as that observed by Wang and colleagues in their study of the role of Ank in nonmineralizing hypertrophic chondrocytes derived from the chick tibia growth plate [7]. In their study, however, wild-type *ank *was greatly overexpressed via retroviral transfection and infection using a replication competent retrovirus [7]. Nevertheless, the concomitant increase in *ank *expression (see Figure 3d) and in AP activity was consistent with that observed by Wang and colleagues [7].

Activities of the PPi-generating enzyme NPP were also evaluated in proliferating cells, in the nonmineralizing, hypertrophic cells, and in mineralizing cells that had been transduced with wild-type and mutant *ank *at days 14, 28, and 35, respectively. Figure 4 demonstrates that, in contrast to cells transduced with wild-type *ank *and the P5T mutant and M48T mutant Ank, the P5L mutant cell line consistently exhibited significantly higher NPP activity during the proliferative, the nonmineralizing hypertrophic, and the mineralization phases of differentiation. All of the transduced cells illustrated an increase in NPP activity at day 35 (mineralization) of culture; this observation was consistent with previous reports showing that an increase in AP expression leads to enhanced levels of PC-1, an NPP isoform [24].

**Figure 5:**
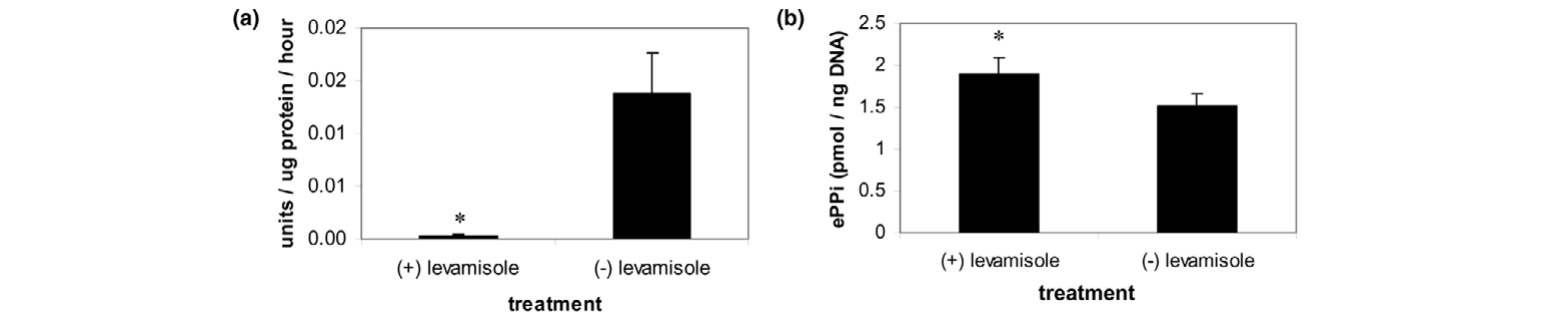
Levamisole treatment of the P5L cell line increases elaboration of extracellular inorganic pyrophosphate. **(a) **Treatment of the P5L-transduced cell line with levamisole results in a dramatic reduction in alkaline phosphatase (AP) activity. **(b) **Treatment with levamisole increases extracellular inorganic pyrophosphate (ePPi) in P5L-transduced cells. *n *= 3; *P ≤ 0.05.

As already discussed, we observed that increased levels of ePPi were not present in the P5L cell line when the cells underwent mineralization, despite the fact that at this stage of differentiation NPP activity was still elevated in the P5L cells. We therefore hypothesized that, during mineralization, high levels of AP activity may have resulted in the hydrolysis of the NPP-driven excess of ePPi in the P5L cell line. To test this hypothesis, the P5L cells were treated with levamisole, an inhibitor of AP activity. Figure 5a,b illustrates that treatment of the P5L cell line with levamisole inhibited AP activity, and resulted in an increase in ePPi in the P5L cells, suggesting that excess ePPi is being hydrolyzed by AP during the mineralization phase. Although depression of AP activity was dramatic, the data suggest that approximately 25% of ePPi was hydrolyzed by AP. These findings are consistent with the trend toward a higher level of ePPi in the P5L cell line during mineralization – although, as discussed above, this trend was not statistically significant (Figure 3c). The failure of AP to further hydrolyze ePPi may relate to the rate of hydrolysis of ePPi by AP in the cell line; however, we did not evaluate the kinetics of ePPi hydrolysis in the current study.

Taken together, our data suggest that at day 35 (mineralizing hypertrophy) neither NPP nor AP are entirely responsible for the high levels of ePPi elaboration among the *ank-*transduced cell lines (Figure 3c), including the P5L cell line. Rather, the results strongly suggest that the increase in ePPi elaboration in the *ank*-transduced cells during the mineralizing hypertrophic stage of chondrogenesis at day 35 is a reflection of the increase in transport activity of Ank (that is, higher levels of *ank *expression).

### Effect of *ank *overexpression, and expression of mutant *ank *on the hypertrophic phenotype in transduced ATDC5 cells

Since studies of idiopathic CPPD disease have suggested that the pathological mineralization of articular cartilage may occur in a matrix that expresses many markers of chondrocyte hypertrophy [5,25], we took advantage of the fact that ATDC5 cells are capable of undergoing a complete course of chondrocyte maturation that would enable us to monitor the course of chondrogenesis in the *ank*-transduced cells. As reported previously [8], the synthesis of *col2a1 *reached maximal levels at day 14 of culture. At 28 days of culture, all cells – whether transduced with empty vector, with wild-type Ank, or with mutant Ank – were morphologically hypertrophic and exhibited a decrease in *col2a1 *and *sox9 *expression levels compared with the levels observed in transduced cells at their proliferative phase (data not shown), with a comcomitant increase in expression of *col10a1 *that peaked at 35 days of culture [8]. These observations showed that stably transduced cells were fully competent to appropriately undergo a course of chondrogenesis, thus indicating that retroviral transduction did not influence the course of chondrogenic differentiation in the cells.

**Figure 6:**
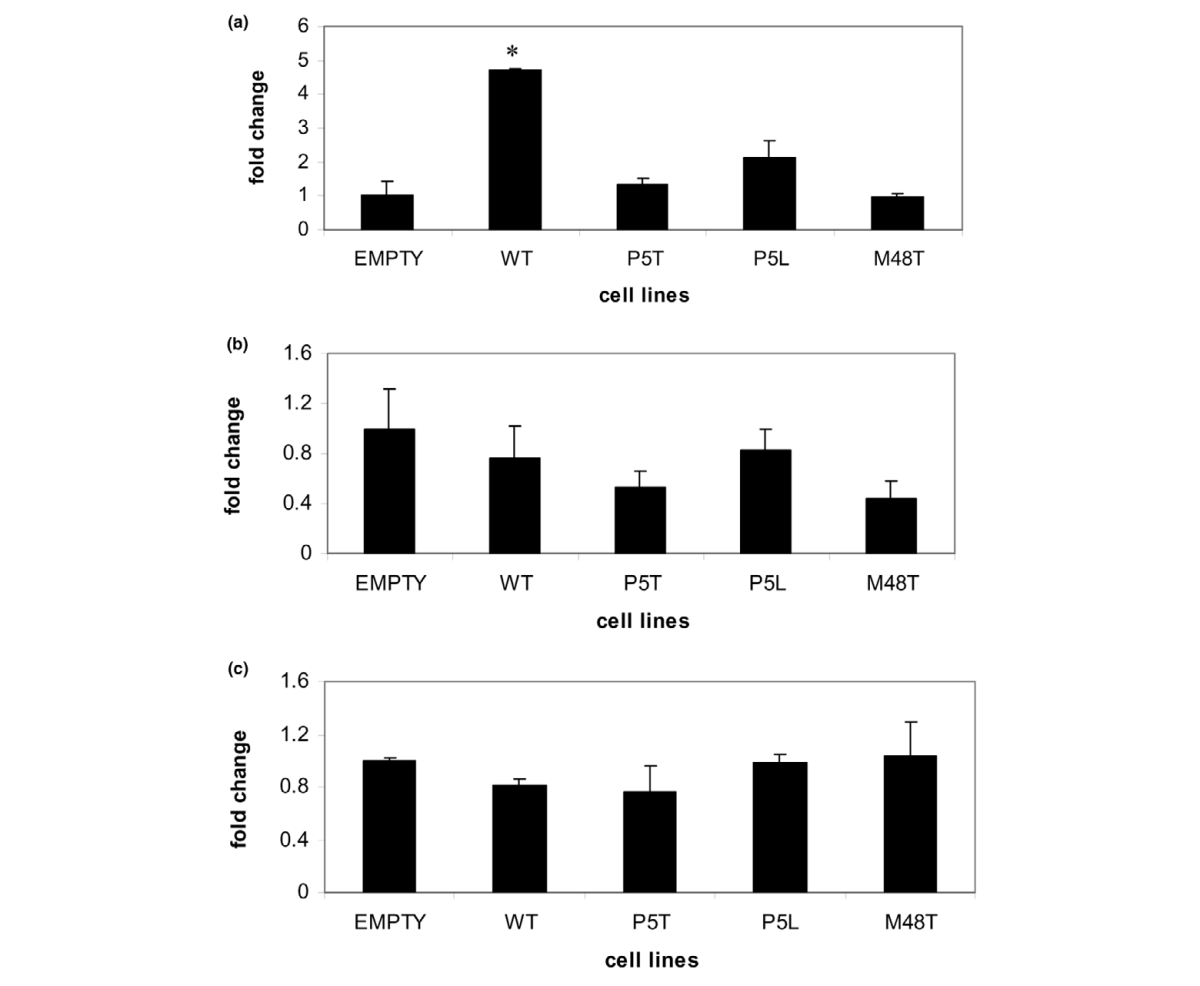
Real-time RT-PCR analyses of hypertrophic markers in transduced lines (day 28) cultured under nonmineralizing conditions. RNA levels were normalized to expression of β-actin. **(a) **Expression of *col10a1 *in transduced lines. Expression is higher than empty vector in cells overexpressing wild-type (WT) *ank *(*P ≤ 0.05); however, in cells expressing mutant *ank*, expression levels of *col10a1 *are equivalent to empty vector. **(b) **Expression of osteopontin in transduced lines. Expression of all transduced cells is lower than empty vector, but not significantly different among cell lines that express mutant *ank *versus cell line overexpressing WT *ank*. **(c) **Expression of *runx2 *(*cbfa1*) in transduced lines. Expression is equivalent for all transduced lines and not significantly different from the expression of *runx2 *in cells transduced with empty vector only. *n *= 6. At least three independent clones for each cell line were evaluated; results presented are from single clones and are representative of other clones for each cell line.

To determine whether the expression of mutant *ank *affected the course of chondrocyte maturation in the transduced cells, we performed real-time RT-PCR to assess the expression of markers of the hypertrophic phenotype in nonmineralizing hypertrophic cells. We examined expression levels for *col10a1*, the classic extracellular matrix marker of hypertrophic chondrocytes, for osteopontin, a secreted glycoprotein that is a marker of terminal differentiation [26,27], and for *runx2 *(*cbfa1*), a transcription factor that is expressed in prehypertrophic and hypertrophic chondrocytes [28,29]. *col10a1 *expression was almost threefold greater in cells that overexpressed wild-type *ank *than in cells transduced with empty vector. In cells transduced with mutant *ank *constructs, however, the expression of *col10a1 *was significantly reduced in comparison with cells overexpressing wild-type *ank *and was moderately reduced in comparison with levels expressed by cells that were transduced with empty vector only (Figure 6a). The expression of osteopontin was not significantly different among the transduced cells in nonmineralizing conditions (Figure 6b). Finally, the expression of *runx2 *was essentially the same for cells transduced with either wild-type *ank *or with mutant *ank *constructs, and was not statistically different from the level of *runx2 *expressed by cells transduced with empty vector only (Figure 6d). Taken together, these results suggest that most markers of the hypertrophic phenotype such as AP, osteopontin, and *runx2 *are unaffected by expression of mutant *ank*, although overexpression of wild-type *ank *resulted in an increase in the expression of *col10a1*.

### Mineralization in ATDC5 cells transduced with wild-type and mutant *ank*

To determine whether transduced cells cultured under mineralizing conditions were competent to undergo mineralization, cells were cultured in a mineralization medium in 3% CO_2 _(see Materials and methods)as previously described [8]. At 35 days of culture, the cells were then processed for analysis of the nature of the mineral phase deposited by all cell lines, including cells expressing mutant Ank molecules. Similar to the cells overexpressing wild-type Ank, the mineral to matrix ratio for cells expressing mutant Ank molecules exhibited low crystallinity compared with those cells transduced with empty vector only (data not shown). In all cases, however, Fourier transform IR analyses of the mineral phase indicated that only basic calcium phosphate was deposited. This finding is probably a result of the high AP activity in the cell lines during mineralization. With respect to the P5L cell line, the hydrolysis of the NPP-driven excess in ePPi by AP is probably also responsible for the deposition of basic calcium phosphate in this cell line.

## Discussion

Recent studies of Ank in a variety of model systems have suggested that the expression of Ank is intimately involved in the regulation of cartilage mineralization and that, at the very least, this regulation includes a triad of constituents: Ank, AP, and isoforms of nucleotide NPP, especially the NPP1 isoform (for a review, see [30]). Human ANK and murine Ank exhibit almost 98% homology at the protein level, with complete conservation of charge and polarity among substituted residues. For this reason, we decided to utilize a mouse cell line for our studies and to prepare mutations in *ank *in the context of the mouse cDNA sequence. Use of the ATDC5 cell line also enabled us to evaluate the effect of overexpression of Ank and expression of mutant Ank on hypertrophy – a point of interest in light of the fact that Uzuki and colleagues recently observed that ANKH immunoreactivity in chondrocytes derived from patients with idiopathic CPPD disease reached maximum levels in areas of affected articular cartilage occupied by chondrocytes exhibiting expression of markers of hypertrophy [5].

In the studies described herein, we chose to only modestly overexpress wild-type and mutant Ank in order to create a stable dominant-negative environment in which to evaluate PPi elaboration, to evaluate AP and NPP activity, and to evaluate expression of markers of hypertrophy in transduced cells. PPi analyses in the Ank mutants indicated that only the P5L mutant generated more ePPi than any other mutant or wild-type Ank transduced cells at the proliferative and nonmineralizing hypertrophic stages of differentiation. This line also consistently displayed greater NPP activity at all stages of maturation than any other cell line. Our studies suggest that the increase in ePPi in the P5L cell line is probably a direct reflection of the NPP activity exhibited by this mutant. These observations are consistent with previous studies showing that Ank regulates PPi levels in coordination with the PPi-generating activity that is specifically contributed by NPP1 [6]. The P5L mutation is unique among the two proline mutations at the 5 position studied here – in that the proline residue is substituted with a neutral, nonpolar amino acid, in contrast to the P5T mutant in which proline is substituted with a polar residue. How this fact may affect the structure of the P5L mutant Ank is presently unclear and must await the studies of Ank transmembrane topology that are currently underway in our laboratory.

We also investigated the steady-state mRNA expression of several hypertrophy-related genes in our ATDC5 cells transduced with wild-type and mutant *ank*. While the course of differentiation appeared to be undisturbed with regard to expression of markers of chondrocyte proliferation and differentiation such as *sox9 *and *col2a1*, we were able to observe subtle differences in the expression of *col10a1 *in the transduced cells that overexpressed wild-type *ank*. Under nonmineralizing hypertrophic conditions, cells overexpressing wild-type *ank *demonstrated levels of expression of *col10a1 *that were twofold to threefold higher than levels in cells transduced with empty vector only. Cells expressing mutant *Ank *constructs, however, did not exhibit any significant change in expression of *col10a1 *when compared with cells transduced with empty vector.

Previous *in vitro *studies of the impact of dominant mutations in ANKH on the function of the ANK protein were performed on transfected COS cells with constructs containing the insertion of four amino acids mutant and the M48T mutant [15]. The familial mutations did not significantly alter iPPi levels, and ePPi levels were not examined. To explain the lack of impact on iPPi observations, it was hypothesized that, in contrast to the *ank*/*ank *recessive mouse mutant, the dominant human CPPD disease mutations might act as gain-of-function alleles that would moderately increase ePPi levels over time, leading to subtle abnormalities that have a minor and cumulative impact on articular cartilage homeostasis [15].

Recent studies of transient transfection of a familial (P5L [17]) ANKH mutation and two ANKH variants (-4 bp, 5' untranslated region [31] and deletion of E490 [15]) in an immortalized human chondrocyte cell line (CH-8) demonstrated an increase in the transcription and translation of ANK. Among the two missense ANKH mutants studied, neither the P5L mutant nor the M48T mutant demonstrated an increase in ePPi elaboration [31]. This effect may, in part, be due to the fact that the studies were performed in an immortalized cell line of human articular chondrocytes that elaborates large amounts of tumor necrosis factor alpha [32], a cytokine reported to inhibit the elaboration of PPi in articular chondrocytes and cartilage explants [33] and shown to dramatically decrease the expression of *ank *mRNA in cultured rat chondrocytes [34]. Interestingly, this same study explored the expression of type X collagen in order to determine whether ANKH variants had the potential to stimulate hypertrophic differentiation in the transfected immortalized articular chondrocytes. Only those cells expressing the P5L variant appeared to induce the expression of type X collagen [31]. Our observations of type X collagen expression show that, among the mutant constructs expressed in transduced cells, the P5L mutant cell line showed the highest level of type X collagen expression; however, all mutant cell lines expressed considerably less *col10a1 *than did cells overexpressing wild-type *ank*.

While the various *in vitro *studies of Ank overexpression on PPi transport activity consistently demonstrate an increase in ePPi levels (or a decrease in iPPi levels) [2,6,7,15,31], there is a notable lack of agreement among studies utilizing the CPPD Ank mutations. This lack of consensus illustrates the limitations of *in vitro *systems, especially with respect to the availability of suitable *in vitro *model systems for the study of diseases of articular cartilage. Future studies of Ank mutations in animal models may illuminate the role of the CPPD-causing mutations on the transport of PPi and the pathological mineralization of articular cartilage.

## Conclusion

In conclusion, we have stably transduced ATDC5 cells with wild-type and mutant cDNA *ank *constructs and have observed that, among the missense mutations, only the P5L mutant resulted in increased activity of NPP and resulted in increased elaboration of ePPi at the proliferative and nonmineralizing hypertrophic stages of differentiation. This effect was tempered during the mineralization phase when AP activity was high in all transduced cells.

We also observed that modest overexpression of *ank *may have subtle effects on chondrocyte maturation. Although this is the first report of a gain-of-function in one of the familial chondrocalcinosis-causing mutations, as previously hypothesized [15], it is not clear why the two other mutations in Ank (P5T and M48T) did not exhibit this effect. The increased ePPi elaboration in the P5L mutant appears to be driven by the excess NPP activity exhibited by this cell line (and other independent cell lines expressing this mutation). This observation may allude to the possibility of increased interaction between the P5L mutant molecule and NPP. The evidence for interaction between NPP and Ank is compelling [6,35,36]. Whether mutations in Ank may alter the affinity of potential interaction between these two proteins is intriguing and warrants further investigation.

It is also not clear at this time whether our observations reflect the state of ePPi elaboration in familial CPPD patients bearing mutations in ANKH. CPPD disease is a disease of articular cartilage that normally does not undergo mineralization, and several reports suggest that the pathological mineralization of articular cartilage in idiopathic chondrocalcinosis may occur in the context of hypertrophic differentiation [5,25]. Interestingly, radiological examination of joints from familial CPPD disease patients indicates that crystal deposition precedes joint degeneration. Consequently, the state of articular cartilage differentiation with respect to markers of hypertrophy is unknown in patients suffering from familial chondrocalcinosis. Our observations of ePPi elaboration during the proliferative and nonmineralizing phases of chondrocyte hypertrophy may therefore hold some relevance in studies of the impact of ANKH mutations in dominantly inherited CPPD disease.

## Abbreviations

*ank *= progressive ankylosis gene/cDNA (murine); Ank = progressive ankylosis protein (murine); ANK = progressive ankylosis protein (human); ANKH = progressive ankylosis gene (human); AP = alkaline phosphatase; bp = base pair; *col2a1 *= gene coding for type II collagen (murine); *col10a1 *= gene coding for type X collagen (murine); CPPD = calcium pyrophosphate dihydrate deposition; DMEM = Dulbecco's modified Eagle's medium; ELISA = enzyme-linked immunosorbent assay; ePPi = extracellular inorganic pyrophosphate; iPPi = intracellular inorganic pyrophosphate; M48T = methionine position 48 to threonine; NPP = nucleotide pyrophosphatase phosphodiesterase; P5L = proline position 5 to leucine; P5T = proline position 5 to threonine; PCR = polymerase chain reaction; PPi = inorganic pyrophosphate; RT = reverse transcriptase.

## Competing interests

The author(s) declare that they have no competing interests.

## Authors' contributions

RZ carried out the preparation of retroviral constructs, the transfections/transduction of cells, the real-time PCR experiments, the PPi assays, and the AP and NPP assays. DS participated in the design of retroviral constructs. ASD purified antibodies and performed the ELISAs. AK made site-directed mutant constructs. FH assisted RZ in the selection and cloning of transduced cell lines. CJW conceived of the study, analyzed and interpreted data, and prepared the manuscript.
